# Clinical and radiographic predictors of acute compartment syndrome in the treatment of tibial shaft fractures: a retrospective cohort study

**DOI:** 10.1186/s12891-020-3044-8

**Published:** 2020-01-13

**Authors:** Lydia Wuarin, Amanda I. Gonzalez, Matthieu Zingg, Patrick Belinga, Pierre Hoffmeyer, Robin Peter, Anne Lübbeke, Axel Gamulin

**Affiliations:** Division of Orthopaedic and Trauma Surgery, University Hospitals of Geneva, 4 Rue Gabrielle-Perret-Gentil, CH-1211 Geneva, Switzerland

**Keywords:** Tibial shaft fracture, Tibial diaphysis fracture, Acute compartment syndrome, Fasciotomy, Risk factors

## Abstract

**Background:**

The purpose of this study was to evaluate the association between epidemiological, clinical and radiographic factors of patients with tibial shaft fractures and the occurrence of acute compartment syndrome.

**Methods:**

270 consecutive adult patients sustaining 273 tibial shaft fractures between January 2005 and December 2009 were included in this retrospective cohort study. The outcome measure was acute compartment syndrome. Patient-related (age, sex), fracture-related (high- vs. low-energy injury, isolated trauma vs. polytrauma, closed vs. open fracture) and radiological parameters (AO/OTA classification, presence or absence of a noncontiguous tibial plateau or pilon fracture, distance from the centre of the tibial fracture to the talar dome, distance between tibial and fibular fracture if associated, and angulation, translation and over-riding of main tibial fragments) were evaluated regarding their potential association with acute compartment syndrome. Univariate analysis was performed and each covariate was adjusted for age and sex. Finally, a multivariable logistic regression model was built, and odds ratios and 95% confidence intervals were obtained. Statistical significance was defined as *p* < 0.05.

**Results:**

Acute compartment syndrome developed in 31 (11.4%) cases. In the multivariable regression model, four covariates remained statistically significantly associated with acute compartment syndrome: polytrauma, closed fracture, associated tibial plateau or pilon fracture and distance from the centre of the tibial fracture to the talar dome ≥15 cm.

**Conclusions:**

One radiological parameter related to the occurrence of acute compartment syndrome has been highlighted in this study, namely a longer distance from the centre of the tibial fracture to the talar dome, meaning a more proximal fracture. This observation may be useful when clinical findings are difficult to assess (doubtful clinical signs, obtunded, sedated or intubated patients). However, larger studies are mandatory to confirm and refine the prediction of acute compartment syndrome occurrence. Radiographic signs of significant displacement were not found to be correlated to acute compartment syndrome development. Finally, the higher rate of acute compartment syndrome occurring in tibial shaft fractures associated to other musculoskeletal, thoraco-abdominal or cranio-cerebral injuries must raise the level of suspicion of any surgeon managing multiply injured patients.

## Background

Tibial shaft fractures represent approximately 2% of all fractures [1]. The AO/OTA classification might be used for their description [2]. Tibial shaft fracture patterns range from low-energy undisplaced fractures to high-energy multifragmented or segmental displaced fractures [3]. The energy delivered to the limb during trauma may also cause soft-tissue lesions, including skin lacerations and acute compartment syndrome (ACS) [4]. Occurrence of ACS in tibial shaft fractures is reported to reach 11.5% [5–8].

At time of assessment by the orthopedic surgeon, patients may be intubated and/or sedated and/or obtunded, and clinical evaluation of ACS (pain out of proportion with the clinical findings and exacerbated by passive muscle stretch) may be impossible to perform [9–13]. In addition to intra-compartmental pressure (ICP) measurement, alternative ACS predictors need to be identified [11, 14, 15].

Research on risk factors for the occurrence of ACS after tibial shaft fractures identified some demographic, injury-related, and clinical predictors, such as young age, male sex, mechanism of injury, high-energy trauma and open fracture [4–8, 16–18]. Three recent studies on potential radiographic predictors of ACS after tibial shaft fractures failed to identify any of them, including AO/OTA classification, fracture length ratio, associated fibular fracture and its distance to the tibial shaft fracture, or segmental tibial shaft fracture [5, 7, 16].

However, some potential radiographic indicators of high-energy trauma in tibial shaft fractures (which might be likely to cause ACS more frequently) have not been studied yet. These potential risk factors include: 1) the displacement (angulation, translation and over-riding) of the tibial shaft fracture (it was hypothesized that higher displacement was associated with higher-energy trauma); 2) the location of the fracture within the tibial diaphysis (it was hypothesized that a trauma producing a more proximal fracture was delivering more energy within a bulkier muscle environment); and 3) the presence of an associated non-contiguous plateau or pilon fracture (it was hypothesized that a second fracture complex separated from the tibial shaft fracture and located either in the proximal or distal metaphysis was a sign of a higher-energy trauma causing two distinct fracture patterns) [19].

To the authors’ knowledge, these potential risk factors were not simultaneously evaluated to date. The objective of this study was therefore to analyze the relation between key demographic, injury-related, clinical and radiographic factors in patients with tibial shaft fractures and the subsequent development of ACS.

## Methods

### Ethics statement

Before starting the study, approval was delivered by the institutional research ethics committee.

### Study population and design

The setting of this retrospective cohort study was a 1900-bed urban academic medical centre delivering primary to tertiary care to 500,000 inhabitants.

The institutional hospitalization diagnoses database was used to identify all consecutive patients admitted with a diaphyseal tibia fracture. Inclusion criteria were: 1) a diaphyseal tibia fracture as defined by the AO/OTA classification code 42 [2], with or without metaphyseal involvement or extension to the knee or ankle joint, and with or without associated fibular fracture; 2) trauma as the cause of fracture; 3) hospital admission between January 2005 and December 2009; 4) definitive treatment in the authors’ institution; and 5) age > 16 years old. At this point, 297 patients with 301 fractures were identified. Exclusion criteria were: 1) hospital admission more than 24 h after initial trauma (no occurrence); 2) pathological fracture (no occurrence); 3) periprosthetic or peri-implant fracture (five fractures); 4) open growth plates (one fracture); 5) transfer to another institution for definitive treatment (one fracture); 6) above or below knee amputation within the first 24 h after the trauma (no occurrence); 7) death within the first 24 h after the trauma (no occurrence); and 8) radiographic images incomplete for full evaluation and measurements (e.g. only one incidence available; 21 fractures).

**Fig. 1:**
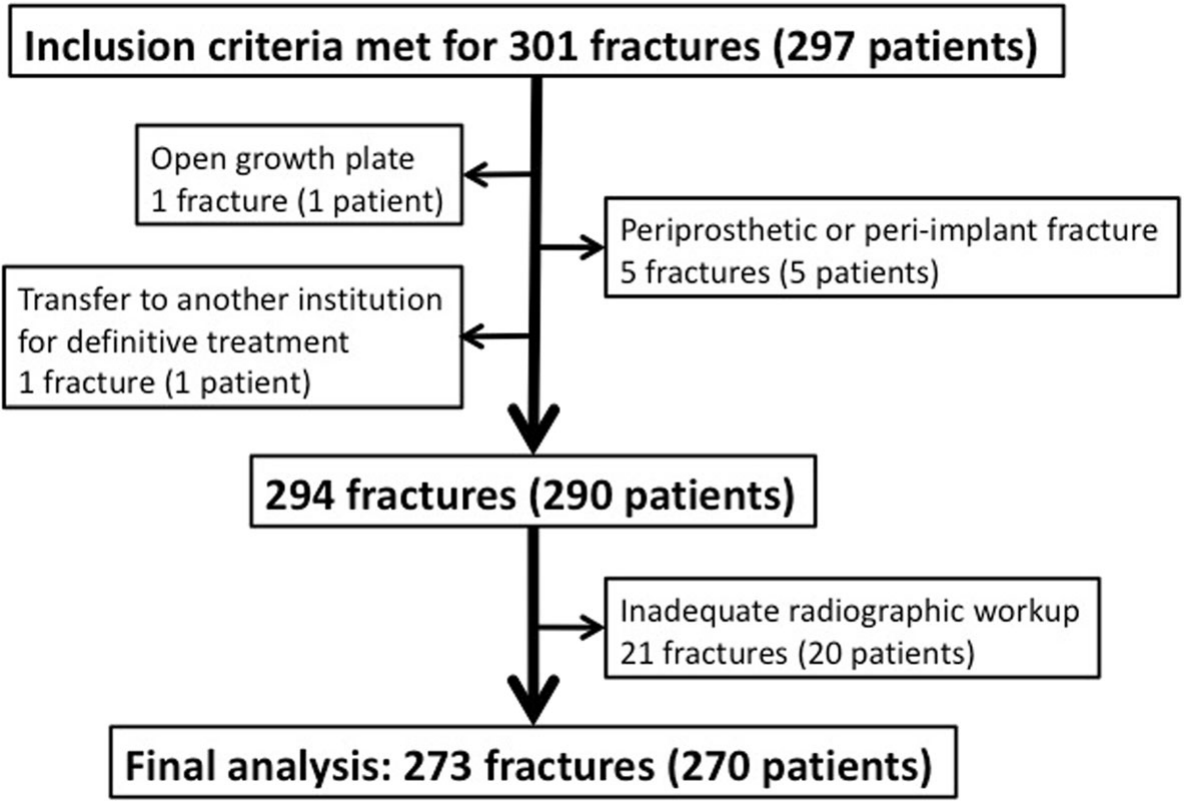
Flowchart depicting patients and fractures meeting inclusion and exclusion criteria, with final numbers available for analysis

Finally, 270 patients with 273 fractures were included in the analysis (Fig. 1). The 21 cases with incomplete radiological data were comparable to the 273 studied cases regarding sex, mean age and incidence of ACS (Table 1).

**Table 1:** Incidence of ACS, sex, and age compared between the population of 270 patients with 273 fractures included in the final analysis and the population of 20 patients with 21 fractures meeting exclusion criteria due to incomplete available radiographic workup

	Final analysis 273 fractures (270 patients)	Patients excluded due to incomplete radiographic workup 21 fractures (20 patients)	*P* value
Male sex, *n* (%)	201 (73.6%)	14 (66.7%)	0.456^a^
Age, mean ± SD	39.9 ± 15.4	42.6 ± 16.6	0.441^b^
ACS occurrence, *n* (%)	31 (11.4%)	2 (9.5%)	1.000^a^

*ACS* acute compartment syndrome

^a^ Fisher’s exact test

^b^ T-test

### Outcome

The outcome was occurrence of ACS leading to fasciotomy. Using the same method as in a previously published study, ICP measurements were not routinely performed to diagnose ACS [19]. When standard clinical evaluation was equivocal (patients with unclear clinical signs and those intubated, sedated or obtunded), ICP was measured in each of the four leg compartments using an Intra-Compartmental Pressure Monitor (Stryker Osteosynthesis AG, Biberist, Switzerland) or an arterial line transducer system with a beveled 18-gauge needle [20]. When ICP was not measured, ACS was confirmed only if muscle bulging or suffering was reported in the operative notes. If muscle was not described in the operative notes, ACS was confirmed only if ICP measurements were pathological.

### Variables of interest

Patients’ charts were reviewed to extract information on age, sex, injury mechanism, isolated trauma or polytrauma, closed or open fracture and Gustilo classification [21, 22] in case of open fractures. In order to distinguish low- and high-energy trauma, injury mechanism was further classified as either “fall from own height” or “other” (falls from more than one meter high, sports or fight injuries, traffic accidents, crushes, construction site accidents). For statistical analysis, fractures were considered in two ways (closed or open) and in three categories (closed, Gustilo type 1, and Gustilo type 2 and 3).

**Fig. 2:**
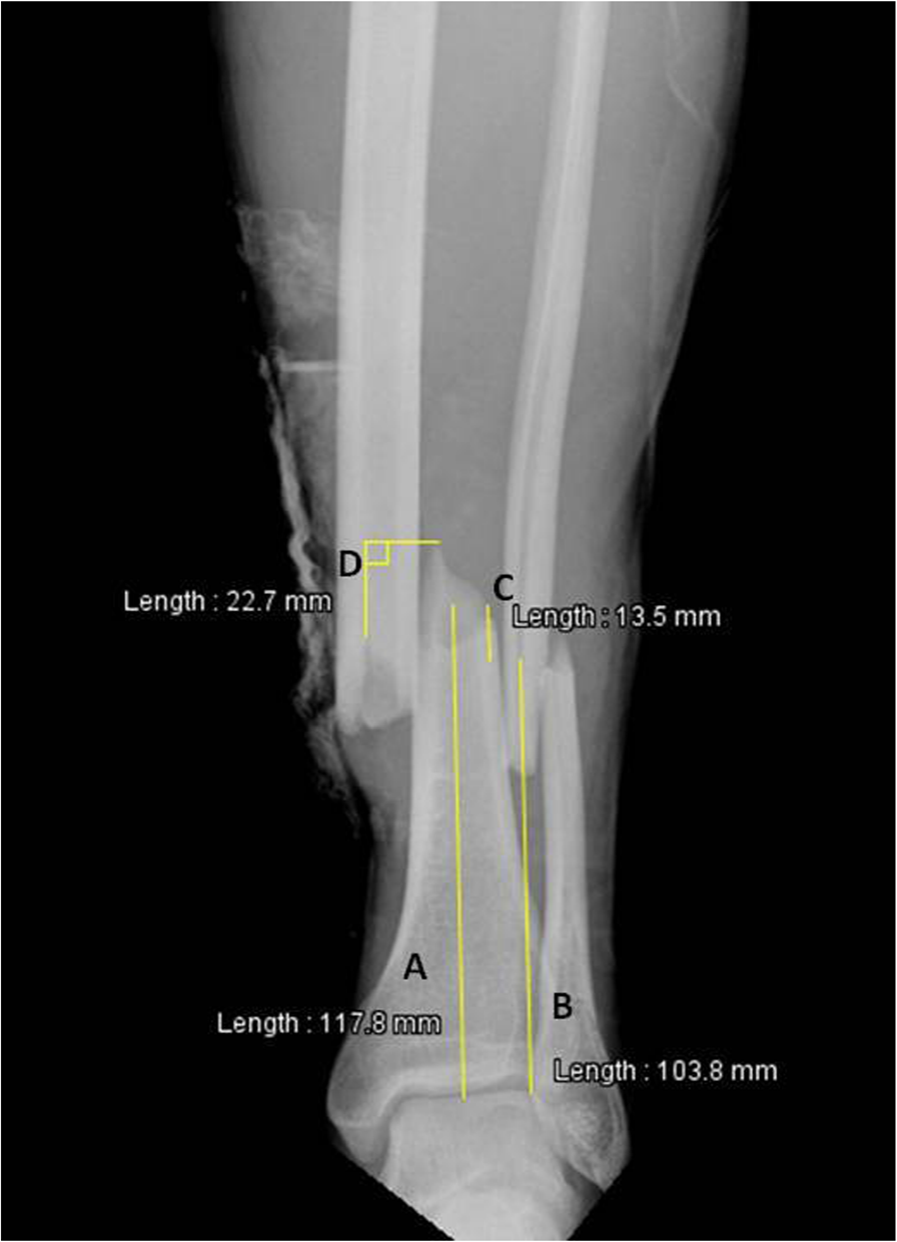
Plain antero-posterior radiograph of a left distal leg including the ankle, with an AO/OTA 42 A2.3 fracture, in a 33.5 years old male patient. Line A represents the distance from talar dome surface to middle of tibial fracture (118 mm in this case); the proximal and distal ends of the tibial fracture were determined taking into account potential intermediate fragments, and the midpoint of the line linking these both points was defined as the center of the tibial fracture (this step is not represented on the figure). Line B represents the distance from talar dome surface to middle of fibular fracture (104 mm in this case); the proximal and distal ends of the fibular fracture were determined taking into account potential intermediate fragments, and the midpoint of the line linking these both points was defined as the center of the fibular fracture (this step is not represented on the figure). In this example, a third spike fragment located at the posterior border of the proximal main fragment and visible on the lateral radiograph (Fig. 4) was present, and its position was anticipated when drawing the proximal and distal end points of the fracture on the antero-posterior radiograph; for this reason, the center of the fibular fracture seems close to what appears to be the distal end of the fracture on the antero-posterior radiograph. Line C represents the distance from tibial fracture to fibular fracture (14 mm in this case). Line D represents over-riding of tibial fracture, which is measured between two corresponding bony landmarks on both main fragments (23 mm in this case)

**Fig. 3:**
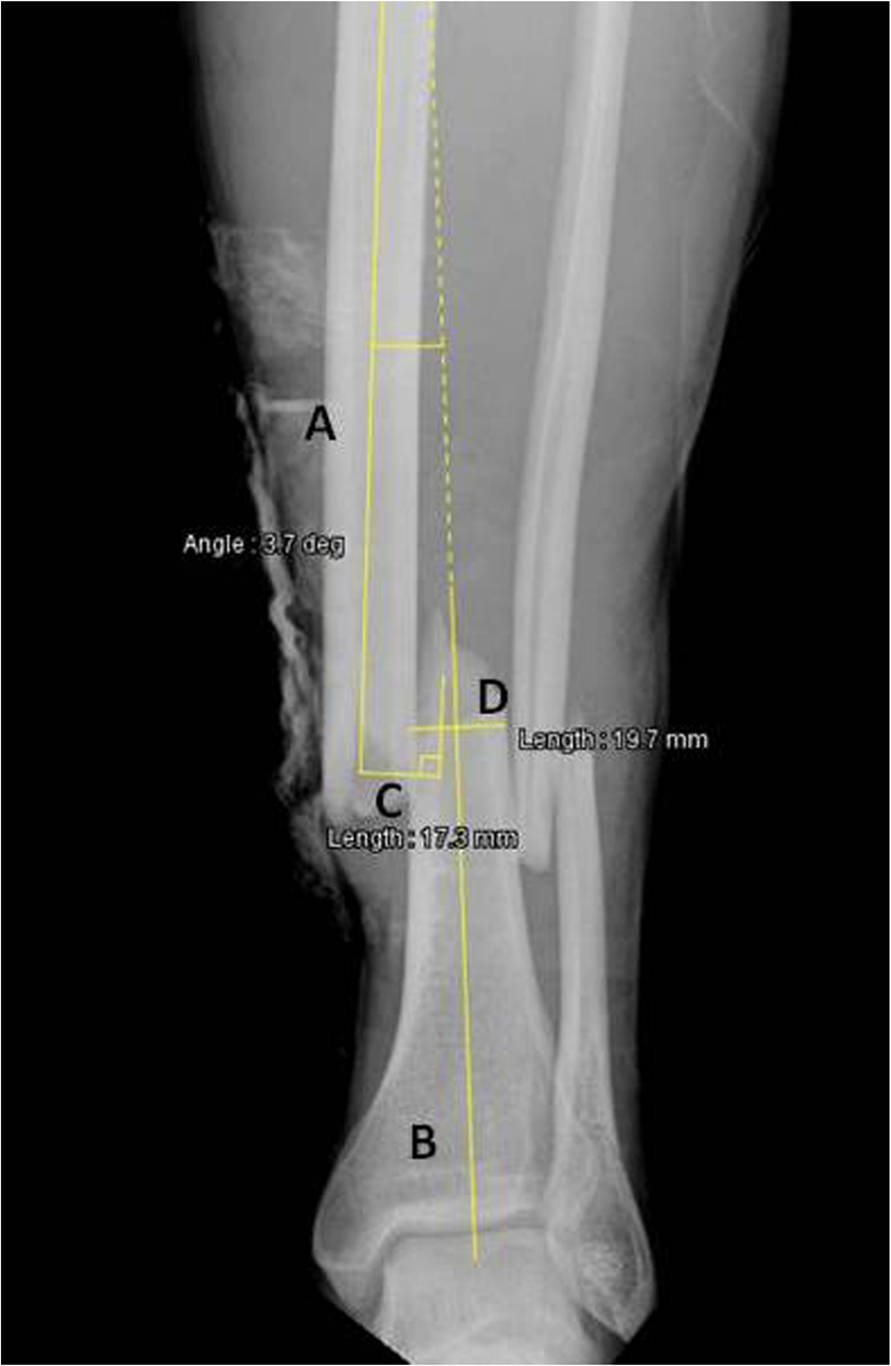
Plain antero-posterior radiograph of a left distal leg including the ankle, with an AO/OTA 42 A2.3 fracture, in a 33.5 years old male patient. Line A represents the anatomical axis of the proximal tibial fragment (the line was drawn in the center of the diaphysis), and line B the anatomical axis of the distal tibial fragment (the line was drawn from the middle of the tibial plafond/talar dome to the center of the diaphysis). Line C represents translation at the level of the fracture center, relative to the axis of the proximal fragment. Line D represents the diameter of the tibia at the level of the fracture. Translation is expressed as a ratio relative to the tibial diameter using the formula Line C/Line D; in this case, 17.3/19.7 = 0.88. Angulation is measured between Lines A and B; in this case, 3.7°

**Fig. 4:**
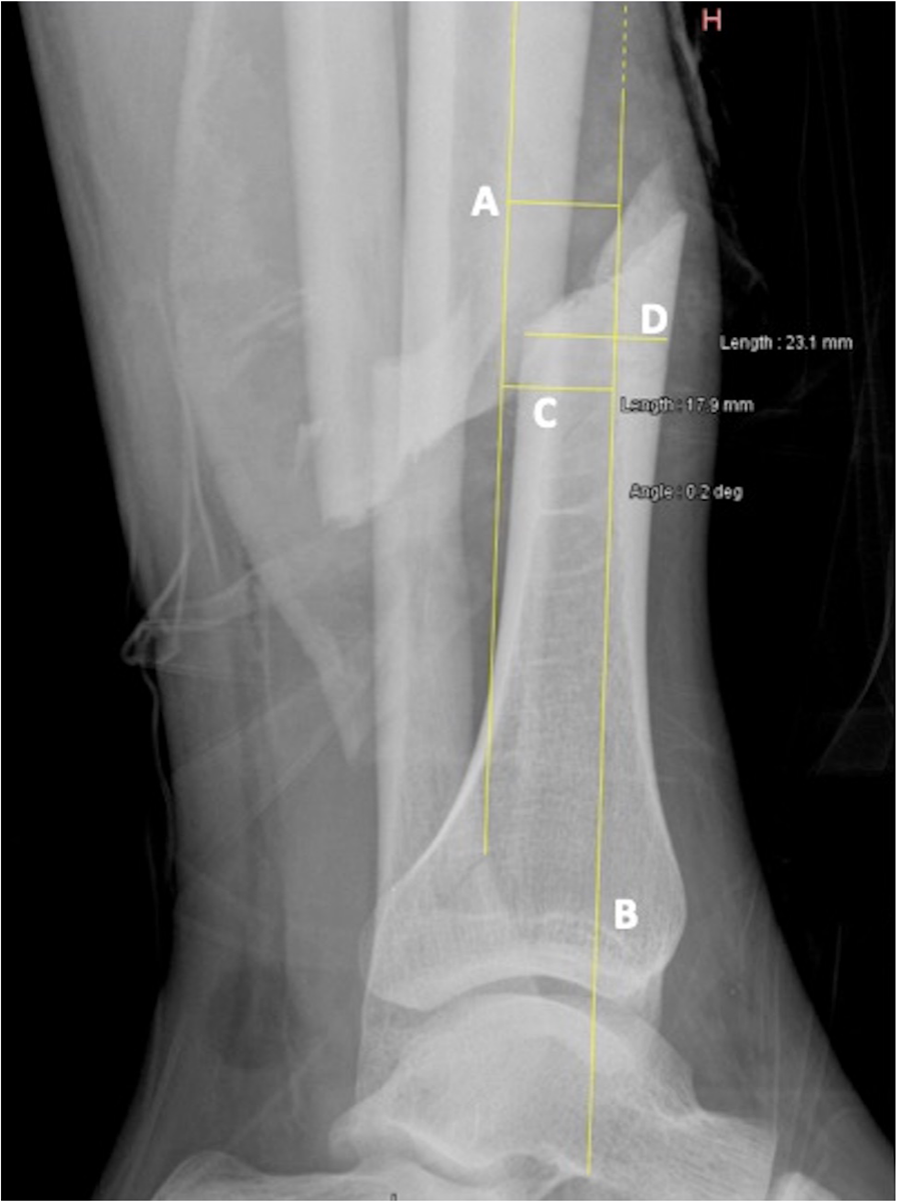
Plain lateral radiograph of a left distal leg including the ankle, with an AO/OTA 42 A2.3 fracture, in a 33.5 years old male patient. Line A represents the axis of the proximal tibial fragment, and line B the axis of the distal tibial fragment; these lines are tangential to the inner or outer border of the anterior cortex, depending on which one of these both borders is the more recognizable on radiograph. Line C represents translation at the level of the fracture center, relative to the axis of the proximal fragment. Line D represents the diameter of the tibia at the level of the fracture. Translation is expressed as a ratio relative to the tibial diameter using the formula Line C/Line D; in this case, 17.9/23.1 = 0.77. Angulation is measured between Lines A and B; in this case, 0.2°

Radiographic evaluation of all cases was performed using a dedicated descriptive form including detailed illustrations of classification schemes and radiographic measurements. Based on the initial set of radiographs, AO/OTA classifications were determined, as well as the presence or absence of a non-contiguous tibial plateau or pilon fracture. Only the first level of the AO/OTA classification (42-A, B and C) was used for the statistical analysis, as inter-observer variability concerning the sub-classification 1, 2 and 3 remains a concern [23]. Non-contiguous tibial plateau or pilon fractures were defined as a second fracture complex separated from the tibial shaft fracture and located either in the proximal (AO/OTA 41 classification) or distal (AO/OTA 43 classification) metaphysis [2]. Radiographic measurements were performed on the initial antero-posterior and lateral plain radiographs of the leg after gross realignment of the limb (as per institutional policy) using a dedicated web-based open-source PACS workstation DICOM viewer (Weasis medical viewer, available on https://nroduit.github.io/en/). A descriptive chart was used to perform the analysis in a reproducible way (Figs. 2, 3 and 4). The following measures were obtained: 1) distance from talar dome surface to middle of tibial fracture in mm (Fig. 2; the proximal and distal ends of the tibial fracture were determined taking into account potential intermediate fragments, and the midpoint of the line linking these both points was defined as the center of the tibial fracture); 2) distance from talar dome surface to middle of fibular fracture in mm (Fig. 2; the proximal and distal ends of the fibular fracture were determined taking into account potential intermediate fragments, and the midpoint of the line linking these both points was defined as the center of the fibular fracture); 3) distance from tibial fracture to fibular fracture in mm, which was measured and also calculated by substracting value of point 1 from value of point 2 (Fig. 2): the mean value between measured distance and calculated distance was used for the analysis; 4) over-riding of tibial fracture in mm (Fig. 2); 5) translation at the level of the fracture center as a ratio relative to the tibial diameter on the antero-posterior incidence (Fig. 3); 6) translation at the level of the fracture center as a ratio relative to the tibial diameter on the lateral incidence (Fig. 4); 7) total translation ratio which was calculated with the formula.

$$ \sqrt{{\left(\mathrm{value}\ \mathrm{of}\ \mathrm{point}\ 5\right)}^2+{\left(\mathrm{value}\ \mathrm{of}\ \mathrm{point}\ 6\right)}^2}; $$

8) angulation on antero-posterior incidence (Fig. 3; the angle was measured between the anatomical axis of the distal fragment, and the anatomical axis of the proximal fragment); 9) angulation on lateral incidence (Fig. 4; the angle was measured between lines tangential to the inner or outer border of the anterior cortex of each main fragment, depending on which one of these both borders was the more recognizable on radiographs); 10) and total angulation, which was calculated with the formula.

$$ {\tan}^{-1}\sqrt{{\mathit{\tan}}^2\left(\mathrm{value}\ \mathrm{of}\ \mathrm{point}\ 8\right)+{\tan}^2\left(\mathrm{value}\ \mathrm{of}\ \mathrm{point}\ 9\right)}. $$

The choice of talar dome instead of tibial plafond to measure the distance to the tibial and fibular fractures was driven by three reasons: a common reference independent from both bones involved by the fracture (tibia and fibula) was preferred; some fractures of the tibial shaft can extend into the tibial plafond and potentially distort its radiological anatomy thus making the choice of a measurement reference on its surface difficult; as initial radiographs were assessed, measurements could be performed before any realignment or external fixation potentially provoking joint space distraction and artificial distance increase.

Radiographic analysis of all cases was first performed by a single surgeon, who was blinded to the outcome (LW, 2 years of experience in orthopedic radiographic interpretation and measurements like the above-described ones). In order to address the possible issue of inter-observer reliability, an Internet based random integer generator (www.random.org) was used to randomly select 30 cases among the 273 fractures. As cases were picked independently from each other, duplicates may occur that were excluded from the reliability assessment. A second orthopedic surgeon, which was trauma-fellowship trained, performed complete radiographic analysis on the randomly picked radiographs (AG, more than 15 years of experience in orthopedic radiographic interpretation and measurements like the above-described ones).

In the cases of ACS occurrence, the delay between injury and ACS and between surgery and ACS (before, during or after surgery), the timing of fasciotomy (during first intervention or later), and the compartment pressure measurements as well as the description of the muscle compartments during fasciotomy (bulging, suffering, necrosis) were extracted from patients’ charts.

### Statistical analysis

Clinical characteristics of patients and fractures with and without ACS were compared using frequency distributions for categorical variables and means and standard deviations for continuous variables. Fischer’s exact test was used for categorical variables and t-test for continuous variables. To evaluate the association of the covariates with ACS, we used logistic regression analysis to obtain univariate odds ratios (OR) and 95% confidence intervals (95% CI). For this analysis, approximate midpoints were used to dichotomize continuous variables (age < 45 years, distance talar dome-middle of tibial fracture ≥15 cm, distance fibular fracture-tibial fracture < 3 cm, total angulation ≥5°, total translation ≥40%, and over-riding ≥8 mm).

Each covariate was then separately adjusted for age and sex using multivariable logistic regression analysis, because the risk of ACS may be related to the energy dissipated at the time of injury and younger men may be more prone to sustain high-energy trauma.

Finally, variables with significance < 0.1 were included in a final multivariable logistic regression model, and ORs and 95% CIs were obtained. Statistical significance was defined as *p* < 0.05 in this final model.

Intraclass correlation coefficients (ICC two-way-random) were obtained to quantify the inter-observer reliability of the radiographic analysis. The ICC is a special case of the weighted kappa and has been considered equivalent [24]. Results were interpreted following the Landis and Koch recommendations: κ < 0 = poor agreement, κ 0.0–0.20 = slight agreement, κ 0.21–0.40 = fair agreement, κ 0.41–0.60 = moderate agreement, κ 0.61–0.80 = substantial /good agreement, κ 0.81–1.00 = almost perfect agreement [25].

IBM® SPSS® Statistics version 22.0 and 25.0 software (IBM SPSS, Chicago, IL) was used for statistical analysis.

## Results

All 273 fractures in 270 patients had complete epidemiological and clinical data as well as complete radiographic measurements available for analysis. Overall, ACS developed in 31 (11.4%) cases. Of these 31 cases, 23 had ICP measurement confirmation and eight had operative notes describing either muscle bulging or suffering. There were no operative notes describing muscle necrosis. There was no false positive diagnosis of ACS occurrence, as every patient who underwent fasciotomy had either pathological ICP measurements before fasciotomy or muscle bulging or suffering described at the time of fasciotomy.

Nine patients were diagnosed with ACS preoperatively. Of those, five were treated with intramedullary nailing (IMN), three with external fixation, and one with plate osteosynthesis; in all cases, surgery occurred within 24 h after admission (range one to 19 h), and the first procedure was four compartment fasciotomy, followed by skeletal fixation. Peroperative ACS (ACS diagnosed after completion of osteosynthesis and treated within the same surgical time) occurred in 12 patients (11 IMN and one external fixator); in all cases, surgery was performed within 28 h after admission (range two to 28 h). Finally, 10 ACS were diagnosed after the patient had left the operative theatre following fracture fixation (nine IMN and one external fixation performed between two and 24 h after admission); patients were brought back to the operative theater for four compartment fasciotomy between seven hours and 9 days after initial fixation.

Table 2 shows demographics, injury characteristics and clinical and radiographic factors of the study patients.

**Table 2:** Demographics, injury characteristics, clinical and radiographic factors of the 270 study patients with 273 tibial shaft fractures

	ACS absent *N* = 242 (88.6%)	ACS present *N* = 31 (11.4%)
Age (years), mean ± SD	40.6 ± 15.9	34.4 ± 9.9
Male sex, *n* (%)	174 (71.9%)	27 (87.1%)
High energy trauma, *n* (%)	168 (69.4%)	28 (90.3%)
Polytrauma, *n* (%)	52 (21.5%)	13 (41.9%)
Soft tissue closed, *n* (%)	158 (65.3%)	25 (80.6%)
Gustilo I, *n* (%)	42 (17.4%)	2 (6.5%)
Gustilo II-III, *n* (%)	42 (17.4%)	4 (12.9%)
AO/OTA classification A, *n* (%)	145 (59.9%)	14 (45.2%)
B, *n* (%)	77 (31.8%)	12 (38.7%)
C, *n* (%)	20 (8.3%)	5 (16.1%)
Associated non-contiguous tibial plateau or pilon fracture, *n* (%)	3 (1.2%)	2 (6.5%)
Distance talar dome-middle of tibial fracture (mm), mean ± SD	135.3 ± 59.1	176.8 ± 68.7
Total angulation (°), mean ± SD	6.6 ± 5.0	7.0 ± 5.0
Total translation (%), mean ± SD	46.1 ± 27.3	42.2 ± 29.1
Over-riding (mm), mean ± SD	9.3 ± 7.9	9.1 ± 7.7
Associated fibular fracture, *n* (%)^a^	200 (82.6%)	26 (83.9%)
Distance fibular fracture-tibial fracture (mm), mean ± SD^a^	99.6 ± 81.4	88.3 ± 74.4

*ACS* acute compartment syndrome

^a^ 200 of the 242 cases without ACS had an associated fibula fracture, as well as 26 of the 31 cases with ACS. Altogether, there were 47 cases without associated fibula fracture

In the univariate analysis (Table 3), age < 45 years, male gender, high-energy trauma, polytrauma, closed fracture, associated tibial plateau or pilon fracture and longer distance from the centre of the tibial fracture to the talar dome (≥15 cm, meaning a more proximal fracture) were associated with an increased rate of ACS with a significance level set at *p* < 0.1. After separate adjustment of each variable for age and sex, there were only small changes in significance levels, except for high-energy trauma. The latter association was attenuated after adjustment and no longer statistically significant.

**Table 3:** Association between variables of interest (demographics, injury characteristics and clinical and radiographic factors) and the occurrence of acute compartment syndrome: univariate analysis and after adjustment for age and sex

	Univariate analysis		Each variable adjusted for age and sex	
	OR (95% CI)	*P* value	OR (95% CI)	*P* value
Age < 45 years.	3.30 (1.23; 8.90)	0.018	–	–
Male sex	2.64 (0.89; 7.82)	0.080	–	–
High energy trauma	4.11 (1.21; 13.95)	0.023	2.76 (0.78; 9.77)	0.117
Polytrauma	2.64 (1.21; 5.74)	0.014	2.29 (1.03; 5.05)	0.041
Closed fracture	2.22 (0.87; 5.61)	0.094	2.57 (0.99; 6.64)	0.051
AO/OTA types B and C	1.82 (0.86; 3.85)	0.121	1.63 (0.75; 3.51)	0.215
Associated tibial plateau or pilon fracture	5.49 (0.88; 34.26)	0.068	5.24 (0.77; 35.46)	0.090
Distance talar dome-middle of tibial fracture ≥15 cm	4.40 (2.02; 9.58)	< 0.001	3.54 (1.59; 7.87)	0.002
Total angulation ≥5°	1.29 (0.60; 2.77)	0.520	1.15 (0.53; 2.53)	0.725
Total translation ≥40%	0.77 (0.36; 1.63)	0.493	0.87 (0.40; 1.89)	0.721
Over-riding ≥8 mm	0.97 (0.46; 2.04)	0.928	1.03 (0.48; 2.22)	0.946
Associated fibular fracture	1.09 (0.39; 3.01)	0.865	1.49 (0.53; 4.23)	0.446
Distance fibular fracture-tibial fracture < 3 cm	0.97 (0.39; 2.45)	0.951	1.20 (0.46; 3.08)	0.712

*OR* odds ratio, *95% CI* 95% confidence interval

In the final multivariable regression model (Table 4), four covariates remained statistically significantly associated with ACS: polytrauma, closed fracture, associated tibial plateau or pilon fracture and distance from the centre of the tibial fracture to the talar dome ≥15 cm.

**Table 4:** Association between variables of interest (demographics, injury characteristics and clinical and radiographic factors) and the occurrence of acute compartment syndrome: multivariable analysis

	Multivariate analysis	
	OR (95% CI)	*P* value
Age < 45 years.	2.26 (0.78; 6.55)	0.132
Male sex	2.61 (0.80; 8.52)	0.112
Polytrauma	2.58 (1.02; 6.49)	0.045
Closed fracture	5.47 (1.73; 17.32)	0.004
Associated tibial plateau or pilon fracture	8.80 (1.03; 75.02)	0.047
Distance talar dome-middle of tibial fracture ≥15 cm	3.49 (1.47; 8.27)	0.005

*OR* odds ratio, *95% CI* 95% confidence interval

Inter-observer reliability showed almost perfect agreement for complete and first level AO/OTA classification, distance talar dome - middle of tibial fracture, total angulation and total translation, and substantial or good agreement for over-riding (Table 5). Of note, two duplicates were excluded from the 30 randomly picked cases, leaving 28 cases (10.3% of 273 cases) for the final analysis.

**Table 5:** Inter-observer reliability assessment of radiographic analysis

	Intraclass correlation coefficient (95% CI)
AO/OTA classification, complete	0.876 (0.750–0.941)
AO/OTA classification, first level (42-A, B and C)	0.927 (0.849–0.966)
Distance talar dome-middle of tibial fracture	0.982 (0.961–0.991)
Total angulation	0.944 (0.882–0.974)
Total translation	0.917 (0.827–0.961)
Over-riding	0.734 (0.490–0.871)

Intraclass correlation coefficient; values < 0 represent poor agreement, values between 0.0 and 0.20 slight agreement, values between 0.21 and 0.40 fair agreement, values between 0.41 and 0.60 moderate agreement, values between 0.61 and 0.80 substantial or good agreement, and values between 0.81 and 1.00 almost perfect agreement. *95% CI* 95% confidence interval

## Discussion

This study highlighted an 11.4% risk of ACS occurrence during the treatment of tibial shaft fractures. Higher distance from the talar dome to the centre of the tibial fracture, associated tibial plateau or pilon fracture, closed fracture and polytrauma were individually confirmed by the multivariable regression analysis as significantly associated with ACS.

Reported incidence rates of ACS in the treatment of tibial shaft fractures range from 3 to 11.5% and seem to be stable over the past decades [5–8, 16]. Our results fall within this range. In this aspect, our study population represents a rather usual cohort of tibial shaft fracture patients. We did not find any clinical records suspect of late ACS sequellae in the charts of patients that were not diagnosed with ACS, and every patient who underwent fasciotomy had pathological ICP values before fasciotomy, and/or presented muscle bulging or suffering at the time of fasciotomy.

Some ACS developed during or after surgery (external or definitive internal fixation) and the surgical procedure may therefore be partly responsible for these occurrences. However, we believe that surgery only represents an aggravating factor, and that the initial fracture with soft tissue injury is the primary factor for the occurrence of ACS. Indeed, after the initial injury, there may be a certain period of soft tissue vulnerability to further surgical aggression, as recently postulated [19].

In the univariate analysis, patients younger than 45 years were about three times as likely to present with ACS than patients over 45 years. However, the multivariable analysis did not confirm this association as statistically significant. Young age is the most consistently observed independent predictor of ACS occurrence throughout the literature [5, 7, 8, 18, 26]. The reasons for ACS occurring more often in younger individuals are thought to be that younger patients tend to have bulkier muscle and thicker, less yielding fasciae [5, 18]. Thus, any increase in intra-compartmental volume is more likely to lead to a rapid rise in ICP and to ACS.

The univariate analysis showed a trend for male patients to be more likely to develop ACS, but statistical significance was not reached in the multivariable analysis. Literature on this subject is contradictory, with some reports showing male sex to be a risk factor for ACS [18], and some others failing to highlight an association between gender and ACS occurrence [5, 7, 8].

This study failed to point out an association between high-energy trauma and development of ACS, although there was a statistically significant association in the univariate analysis. The literature is controversial on this topic, as one study showed an association between high-energy trauma and ACS occurrence [6] while others did not [5, 7, 18]. Indeed, retrospective determination of the amount of energy released during initial trauma might be unreliable, as chart review may not provide a good representation of the detailed mechanism of injury (for example, type of sports injury, vehicle speed, etc.) [19]. In this perspective, our retrospective attempt to differentiate between low and high-energy mechanism by classifying trauma into “fall from own height” and “other” makes any conclusion about energy of the injury weak.

Interestingly, differentiation between isolated trauma and polytrauma seems to be better in determining possible association with ACS development, as polytrauma was found to be associated with ACS occurrence in the multivariable analysis. This differentiation did not use the Injury Severity Score (ISS) [27, 28], but rather relied on a subjective appreciation of the extension of trauma: the fractured leg (tibia and/or fibula) was either an isolated injury or associated to other musculoskeletal, thoraco-abdominal, spine or cranio-cerebral injuries. This subjective appreciation can be quickly performed by the treating surgeon during the initial survey of any trauma patient and is probably more reliable than any anamnestic investigation in determining the potential risk of ACS occurrence. Furthermore, it does not rely on an a posteriori ISS determination, which is usually performed several hours after the trauma [27, 28]. However, to mitigate this statement, it is important to note that despite an increase in high-energy trauma as a causative injury and an improved survival rate among severe polytrauma patients noted over the past decades [29, 30], ACS rates following tibial shaft fractures do not seem to have increased during the same period [5–8, 16].

Despite older reports recognizing open fractures as positively associated with the occurrence of ACS in tibial shaft fractures, with an incidence of ACS directly proportional to the severity of the open fracture [4, 17], our results are more in line with recent publications having not found any association between open fractures and ACS development [5, 7, 8]. Our results even show that open fractures might have a protective effect against ACS development. Importantly, these findings must not lead clinicians to be wrongly reassured by an open fracture, assuming that the wound would relieve the pressure inside the muscle compartments, as ACS may still develop in these occurrences [5–8]. In fact, skin wounds in the vicinity of a fracture should be recognized as a direct sign of increased amount of underlying fascial and muscle injury and must not be underestimated by the treating physician [19].

Although the first level of the AO/OTA classification (42-A, B and C) is supposed to reflect the amount of energy delivered to the bone and surrounding soft tissues to produce the fracture [23], this parameter did not reach statistical significance in the multivariable analysis. There was nevertheless a distinctive trend for higher-grade fractures to develop ACS, with 14 of 159 42-A fractures (8.8%), 12 of 89 42-B (13.5%) and 5 of 25 42-C (20%). Another study found similar results several years ago [7]. Thus, this radiographic parameter should be used with caution in evaluating the potential risk for ACS to occur. The incidence of ACS in the setting of 42-A fractures should not be underestimated or discarded, and the index of suspicion should remain high.

The presence of an associated non-contiguous tibial plateau or pilon fracture was highlighted in this study. It represents a red flag and an indicator of a high amount of energy transmitted to the injured limb, causing increased skeletal lesions (associated non-contiguous tibial plateau or pilon fracture) and extensive soft tissue damage potentially leading to the development of ACS. Similar results were recently published, where the presence of a non-contiguous tibial fracture or knee dislocation was a predictor of ACS development after tibial plateau fractures [19]. However, this finding is statistically weak, due to low numbers of events (three in the group without ACS and two in the group with ACS) leading to extended CIs.

The most powerful factor highlighted by this study in predicting occurrence of ACS during the treatment of tibial shaft fractures is the distance between the talar dome and the centre of the tibial fracture. In other words, the more proximal the tibial fracture is, the more likely ACS will occur. A previous study found that the incidence of ACS was higher with tibial plateau fractures than with tibial shaft or pilon fractures, but there were no specific results presented on fracture localization within the tibial shaft [16]. This factor has been specifically evaluated only once in the literature, and no influence of the localization of the fracture within the tibial shaft (proximal vs. middle vs. distal third) on the development of ACS could be demonstrated [6]. Our finding is new and may be explained by the fact that a fracture occurring at a location surrounded by a bulkier muscle mass (proximal diaphysis) may lead to more energy transmitted to the soft tissues, thus to the potential development of ACS. This observation may be useful when clinical findings are difficult to assess (doubtful clinical signs, obtunded, sedated or intubated patients). However, the design of the present study did not allow calculating this distance as a ratio relative to the total length of the tibia, which could have been an interesting element to generalize the use of this finding. This could be the aim of a further study on this topic.

One of our initial hypotheses, postulating that higher fracture displacement would be associated to higher-energy trauma and more extended soft tissue damages leading to ACS, could not be confirmed by this study. No association between angulation, translation or over-riding at the fracture site and occurrence of ACS could be demonstrated. As per institutional policy, grossly deformed limbs are aligned by gentle traction and rotational control before any radiographs are taken, in order to protect vascularization and to relieve soft tissue suffering of the injured limb as soon as possible. Thus, radiographs may not reflect the initial deformity and this makes any conclusion about fracture displacement weak.

Additionally, neither the presence or absence of an associated fibular fracture nor the distance between the tibial fracture and the fibular fracture when present showed an association with ACS development. This is in line with previously published data [16].

Despite being one of the largest series to date analyzing the association between key demographic, injury-related, clinical and radiographic parameters in tibial shaft fracture patients and the development of ACS, this study suffers several limitations: 1) the retrospective attempt to differentiate between low and high-energy trauma on chart review makes any conclusions about mechanism or energy of the injury weak; 2) ICP measurements, which would have been the gold standard to diagnose or exclude ACS, were performed only on a subset of patients, thus introducing the possibility of false positive or false negative diagnosis; however, we did not find any clinical records suspect of late ACS sequellae in the charts of patients that were not diagnosed with ACS, and every patient who underwent fasciotomy had pathological ICP values before fasciotomy, and/or presented muscle bulging or suffering at the time of fasciotomy; 3) radiographic analysis was performed on one occasion only thus not quantifying intra-observer variability and measurement imprecision; however, inter-observer reliability assessment performed on 10.3% of the cases showed almost perfect agreement for all measurements, except for over-riding which had substantial or good agreement: this may validate the entire radiographic measurement process performed by an observer with 2 years of experience in orthopedic radiographic interpretation and measurements, as the observer who took part in the inter-observer reliability assessment had more than 15 years of experience in the field; 4) the most powerful factor highlighted by this study in predicting occurrence of ACS during the treatment of tibial shaft fractures, namely the distance between the talar dome and the centre of the tibial fracture, was measured as a distance rather than as a ratio relative to the total length of the tibia; the design of the present study did not allow such a measurement, and this ratio determination could be the aim of a further study in order to generalize the use of this finding; 5) and finally, proximal tibiofibular dislocation, which has been recently shown to be possibly associated with a higher rate of ACS (29%) in both tibial plateau and tibial shaft fractures, was not investigated in the present study [31].

## Conclusions

One radiological parameter related to the occurrence of ACS has been highlighted in this study, namely a longer distance from the centre of the tibial fracture to the talar dome, meaning a more proximal fracture within the tibial shaft. A potential explanation is that a fracture occurring at a location surrounded by a bulkier muscle mass (proximal diaphysis) may lead to more energy transmitted to the soft tissues, thus to the potential development of ACS. This observation may be useful when clinical findings are difficult to assess (doubtful clinical signs, obtunded, sedated or intubated patients). However, larger studies are mandatory to confirm and refine the prediction of ACS occurrence, and to develop this finding into an easily usable ratio relative to the total length of the tibia. Radiographic signs of major displacement were not found to be correlated to ACS occurrence and can therefore not be used in the assessment of ACS risk in case of tibial shaft fracture. Finally, the higher rate of ACS occurring in tibial shaft fractures when associated to other musculoskeletal, thoraco-abdominal, spine or cranio-cerebral injuries must raise the level of suspicion of any surgeon managing multiply injured patients.

## Data Availability

The datasets used and/or analyzed during the current study are available from the corresponding author on reasonable request.

## Abbreviations

95% CI: 95% confidence intervals
ACS: Acute compartment syndrome
AO/OTA: Arbeitsgemeinschaft für Osteosynthesefragen / Orthopaedic Trauma Association
DICOM: Digital imaging and communications in medicine
ICP: Intra-compartmental pressure
IMN: Intramedullary nailing
ISS: Injury severity score
OR: Odds ratios
PACS: Picture archiving and communication system
